# Une tumeur exceptionnelle du doigt: la tumeur fibreuse solitaire

**Published:** 2011-12-01

**Authors:** Taoufiq Harmouch, Nawal Hamas, Abdelkrim Daoudi, Afaf Amarti

**Affiliations:** 1 Laboratoire d'anatomie et de cytologie pathologiques, CHU Hassan II, Fès, Maroc; 2 Service de traumatologie orthopédie, CHU Hassan II, Fès, Maroc

**Keywords:** Tumeur fibreuse solitaire, tissus mous, doigt, immunohistochimie, Maroc

## Abstract

La tumeur Fibreuse solitaire (TFS) est une prolifération fusocellulaire rare, souvent de localisation intra-thoracique. Nous rapportons le cas d'une localisation exceptionnelle au niveau du 3^ème^ doigt de la main droite. En l'absence de marqueur spécifique l'interprétation de l'immuno-marquage dépend du contexte histo-morphologique. La similitude avec les différents néoplasmes à cellules fusiformes des tissus mous rend le diagnostic de ces tumeurs mésenchymateuses difficile et tardif. Dans 20à 30% des cas le comportement de la TFS est celui d'une tumeur maligne localement agressive et récidivante, avec des métastases rares et tardives. Ce comportement impose une surveillance prolongée après exérèse chirurgicale.

## Introduction

La tumeur fibreuse solitaire est une tumeur rare d'origine mésenchymateuse faite d'une prolifération de cellules fusiformes. Elle a été décrite pour la première fois par Klemperer et Rabin au niveau de la plèvre, et anciennement connue sous les noms mesothélima fibreux bénin, fibrome sous-mésothélial, mésothéliome fibreux localisé, ou fibrome pleural. Récemment, des localisations extra-thoraciques ont été décrites notamment au niveau de la tête et du cou, l'abdomen, le rétropéritoine, l'orbite, les voies aériennes supérieures et les tissus mous [1–4]. Nous rapportons une nouvelle observation d'une tumeur fibreuse solitaire bénigne des tissus mous avec une localisation exceptionnelle au niveau de la main.

## Observation

**Figure 1:**
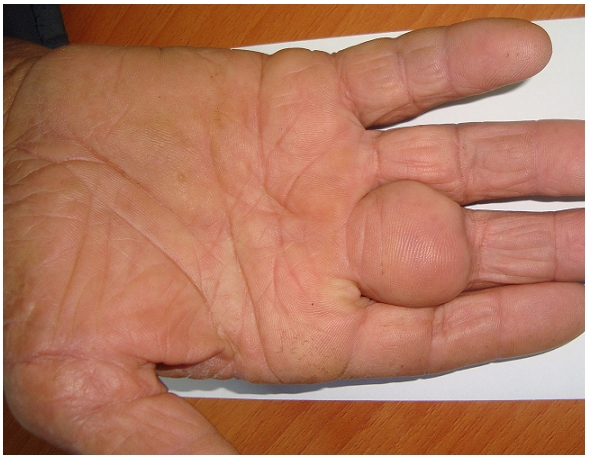
Tumeur de 2,5 cm de grand axe, intéressant la partie proximale du troisième doigt de la main droite

Patient âgé de 62 ans, sans antécédents pathologiques notables, a consulté pour une masse du troisième doigt de la main droite, évoluant depuis 5 mois, indolore et sans impotence fonctionnelle. L'examen clinique a montré la présence d'une masse de 2,5 cm de grand axe, molle, indolore et mobile par rapport au plan profond. La peau en regard est strictement normale (Figure 1).

Une échographie a montré la présence, au niveau du plan graisseux sous cutané, d'un processus tissulaire de 20mm de grand axe, hypervasculaire, hétérogène, hypoéchogène, et à limites nettes. Les structures musculaires et osseuses sont respectées.

**Figure 2:**
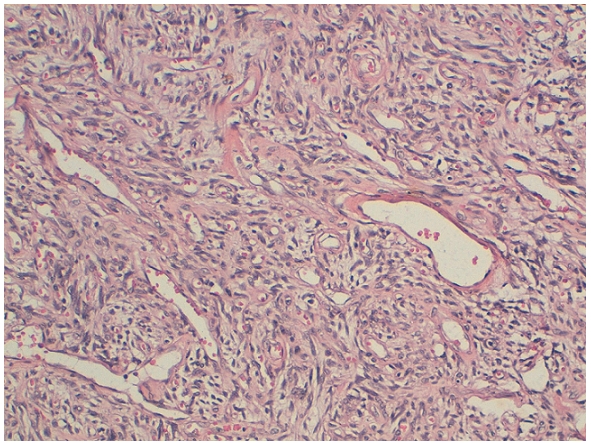
Prolifération tumorales faite de cellules fusiformes au sein d'un stroma riche en vaisseaux à paroi hyalinisée et l'allure hémangiopéricytaire (Coloration standard HES, GX200)

La tumeur est réséquée en totalité. L'examen macroscopique retrouve une masse arrondie, bien limitée, grisâtre et homogène. L'examen histologique montre une prolifération tumorale nodulaire, bien limitée mais non encapsulée, dissociée par endroit par des bandes de collagène. La prolifération tumorale est faite de cellules fusiformes sans atypies cytonucléaires ni foyer de nécrose. Les mitoses sont estimées à 3 mitoses par 10 champs au fort grossissement. La vascularisation est branchée de type hémangiopéricytaire avec un aspect hyalinisé des parois vasculaires (Figure 2).

**Figure 3:**
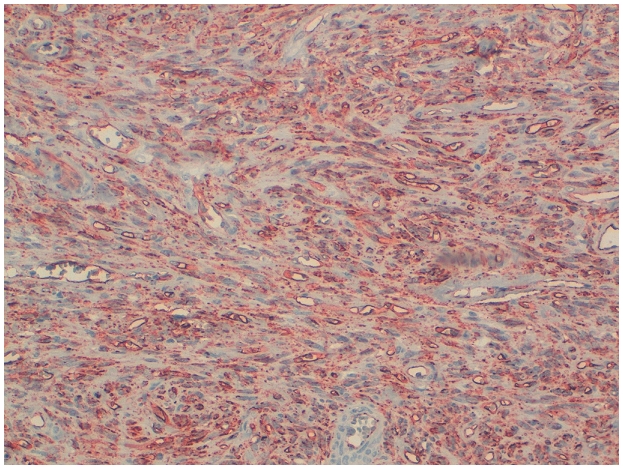
Immunohistochimie: Les cellules tumorales expriment de façon diffuse et intense l'antigène CD34 (GX200)

Les cellules tumorales expriment le CD34 (Figure 3). Les autres marqueurs: CD99, le Bcl2, l'AML, la desmine, la cytokératine et l'EMA ne sont pas exprimés. Le diagnostic retenu est celui d'une tumeur fibreuse solitaire (TFS) bénigne avec des limites saines. Après un recul de 20 mois aucune récidive locale ou métastase à distance ne sont notées.

## Discussion

La TFS est une tumeur rare, survenant chez l'homme et la femme sans prédominance de sexe avec une moyenne d’âge d′environ 57 ans et des extrêmes de 42 et 67 ans [3,5–8]. Cette tumeur a été décrite pour la première fois par Klemperer et Rabin en 1931 comme une tumeur fusocellulaire pleurale [5].

L'origine mésothéliale de cette tumeur n′est plus retenue et il paraît clair actuellement, d'après plusieurs études histologiques et immunohistochimiques, qu'elle soit mésenchymateuse d'origine (myo) fibroblastique [3,9].

Elle est loin d’être une tumeur cantonnée aux séreuses et il est maintenant bien admis qu'elle peut toucher plusieurs localisations extra-pleurales notamment la cavité abdominale, le rétropéritoine, le médiastin, l′orbite, les voies aériennes supérieures et les tissus mous comme c'est le cas rapporté par notre observation. La TFS extrapleurale représente environ 0,6% des tumeurs des tissus mous.

Les manifestations cliniques de la TFS extrapleurale dépendent de son siège. Des signes systémiques comme l'hypoglycémie par insulino-sécrétion, les arthralgies et les ostéoarthrites ont été décrits. Ces symptômes disparaissent habituellement après exérèse de la tumeur [3,7,8].

Macroscopiquement, la tumeur est souvent bien circonscrite, encapsulée, de taille variable allant de 2 à 15cm de diamètre. Cependant, des tumeurs de plus de 30cm ont été rapportées. A la coupe, la tumeur est souvent ferme, blanchâtre et multinodulaire. Des remaniements myxoïde, kystiques ou hémorragiques peuvent être observés. Les foyers de nécrose et de calcifications focales sont inhabituels. Lorsque la tumeur siège au niveau d'une surface séreuse, un pédicule vasculaire est souvent noté [3,6–8].

Sur le plan histologique, la TFS extrapleurale présente le même aspect que la TFS pleurale. Elle se caractérise par une prolifération assez monomorphe de cellules fusiformes, regroupées en faisceaux plus ou moins longs, enroulées, incurvés ou ondulés, disposées sur un fond riche en fibres de collagène. Elles sont dotées d'un noyau arrondi ou ovalaire, parfois fusiforme. Le cytoplasme est peu abondant et les limites cytoplasmiques sont mal définies. Les cellules ne présentent habituellement pas d'atypies cytonucléaires et les mitoses sont rares et habituellement inférieures à quatre pour 10 champs au fort grossissement. La cellularité est variable. Elle est généralement inversement liée au degré de la formation des fibres de collagène. Le réseau vasculaire est très développé et de type hémangiopéricytaire, fait de vaisseaux à paroi fine, ramifiés avec une hyalinisation périvasculaire [1,3,5–7,10,11].

Le profil immunohistochimique est assez évocateur. Il permet de faire le diagnostic différentiel avec les autres tumeurs à cellules fusiformes. La vimentine est toujours exprimée. Le CD34 (glycoprotéine transmembranaire de110 kDa retrouvée à la surface des cellules progénétrices hématopoïétiques et au niveau de l'endothélium vasculaire) est exprimé dans 90-95% des cas. Ce marqueur qui n'est pas spécifique de la TFS et il doit être interprété selon le contexte histomorphologique et les données apportés par les autres marqueurs. Le CD99 et le Bcl2 sont exprimés dans 50% des cas. L'immunomarquage par la béta-caténine et la p53 a été également rapporté [1,3–6,8]. Certaines séries rapportent l'expression focale des récepteurs estroprogestatifs par les cellules tumorales comme facteur de risque favorisant les récidives après exérèse chirurgicale [9].

L′actine musculaire lisse est généralement non exprimée par les cellules tumorales, malgrès l'histogenèse supposée (myo) fibroblastique. Cette contradiction s′explique par le fait qu′il semble y avoir quatre phénotypes (myo) fibroblastiques différents, dont l′un n′exprime pas d′actine [3].

20-30% des cas sont variablement positifs à l'EMA et au Bcl2. Une réactivité focale à la PS100, à la cytokératine et/ou à la desmine ont été occasionnellement rapportés [10].

La plupart des TFS sont bénignes, mais environ 20% d'entre elles peuvent être malignes. La malignité peut survenir de novo ou après une dédifférenciation. Enzinger et Smith ont proposé une combinaison d'une activité mitotique élevée (4 mitoses par 10 champs au fort grossissement), une taille tumorale élevée (>5 cm), une cellularité élevée, la présence de cellules pléomorphes ou immature et la présence de foyers de nécrose et d'hémorragie comme des éléments caractéristiques de la TFS maligne. Néanmoins, les tumeurs qui présentent ces signes histologiques de malignité montrent souvent une évolution clinique bénigne et seulement 50% de ces tumeurs montrent des signes cliniques de malignité comme les métastases à distance ou l'invasion locale [1,7,10].

La TFS peut être difficile à diagnostiquer du fait de sa similitude avec d'autres tumeurs des tissus mous. Le diagnostic différentiel se fait avec les tumeurs fusocellulaires et les pseudotumeurs notamment l'hémangiopéricytome (HPC).

Il faut noter que la vascularisation de type hémangiopéricytaire n'est pas spécifique de la TFS et peut se voir dans plusieurs autres tumeurs [3].

Le diagnostic différentiel avec l'hémangiopéricytome peut être difficile car les deux tumeurs peuvent avoir le même profil immuno-histochimique. Toutefois, l'HPC présente une vascularisation plus riche et tend à présenter un marquage moins intense par le CD34. Récemment, l'hémangiopéricytome a été reclassé comme une variante de la TFS [8].

La fasciite nodulaire, qui est une lésion fibro-myofibroblastique pseudosarcomateuse riche en cellules fusiformes, montre un marquage diffus par l'anticorps anti AML alors que l'anti CD34 est souvent négatif. Le neurofibrome et le schwannome peuvent exprimer le CD34, ils sont également marqués par la PS100. Le myofibrome/myofibromatose et la fibromatose sont souvent fortement et diffusément marqués par la vimentine et l'AML, alors que le CD34 est toujours négatif [10].

La chirurgie est le traitement de choix de la TFS. Une surveillance à long terme est recommandée pour toute TFS du fait de son évolution imprévisible.

La TFS est généralement une tumeur de bon pronostic après résection chirurgicale. Le taux de malignité dans la littérature est de 12-35%. Les séries de TFS intrathoraciques rapportent un taux de récidives locales de 9–19%, un taux de métastases de 0–19% et un taux de décès de 0–27%. Parmi les TFS extrathoraciques, environ 10% semblent être associées à une récidive locale ou à distance. Le facteur de récidive le plus important est l'infiltration des limites de résection chirurgicale [1,3,5,7,11].

## Conclusion

Les TFS des tissus mous notamment au niveau des extrémités sont exceptionnelles. Le diagnostic repose essentiellement sur l'histologie couplée à l'immunomarquage. Le CD34 est un marqueur sensible mais non spécifique des TFS et dont l'interprétation dépend du contexte histomorphologique et de l'expression des autres marqueurs. Malgré son caractère souvent bénin, une surveillance prolongée après exérèse est préconisée devant le risque de récidive.
